# New-onset atrial fibrillation in critically ill acute kidney injury patients on renal replacement therapy

**DOI:** 10.1093/europace/euab163

**Published:** 2021-08-01

**Authors:** Tapio Hellman, Panu Uusalo, Mikko Johannes Järvisalo

**Affiliations:** Kidney Center, Turku University Hospital and University of Turku, Hämeentie 11, PO Box 52, Turku 20521, Finland; Department of Anaesthesiology and Intensive Care, Turku University Hospital and University of Turku, Hämeentie 11, PO Box 52, Turku 20521, Finland; Perioperative Services, Intensive Care and Pain Medicine, Turku University Hospital and University of Turku, Hämeentie 11, PO Box 52, Turku 20521, Finland; Kidney Center, Turku University Hospital and University of Turku, Hämeentie 11, PO Box 52, Turku 20521, Finland; Department of Anaesthesiology and Intensive Care, Turku University Hospital and University of Turku, Hämeentie 11, PO Box 52, Turku 20521, Finland; Perioperative Services, Intensive Care and Pain Medicine, Turku University Hospital and University of Turku, Hämeentie 11, PO Box 52, Turku 20521, Finland

**Keywords:** Atrial fibrillation, Acute kidney injury, Mortality, Continuous veno-venous haemodialysis, Intermittent haemodialysis, Intensive care unit

## Abstract

**Aims:**

The effect of new-onset atrial fibrillation (NOAF) on mortality in critically ill patients with acute kidney injury (AKI) treated in the intensive care unit (ICU) requiring continuous veno-venous haemodialysis (CVVHD) or intermittent haemodialysis (IHD) is unknown. Thus, we examined the incidence of NOAF in critically ill AKI patients undergoing CVVHD or IHD and the association between the timing of NOAF incidence in relation to renal replacement therapy (RRT) initiation and 1-year mortality.

**Methods and results:**

Out of the 733 consecutively recruited ICU patients requiring RRT within the study period of 2010–2019, 516 patients without prior atrial fibrillation history were included in this retrospective study. Clinical comorbidities, medications and biochemistry as well as outcome data for 1-year all-cause mortality were recorded. Episodes of NOAF were collected from the pooled rhythm data covering the entire ICU stay of every patient. The median age was 64 (inter-quartile range 19) years, 165 (32%) were female, and 356 and 160 patients received CVVHD and IHD, respectively. NOAF was observed in 190 (37%) patients during ICU care and 217 (42%) patients died within the 1-year follow-up. Incident NOAF was independently associated with 1-year mortality in the multivariable logistic regression analysis after adjusting for dialysis modality, need for mechanical ventilation or vasopressor support and Acute Physiology And Chronic Health Evaluation II score. However, NOAF diagnosed after RRT initiation was not associated with mortality.

**Conclusion:**

NOAF emerging before RRT initiation is associated with increased mortality in critically ill AKI patients requiring RRT. However, NOAF during RRT does not seem to be associated with mortality.

**What’s new?**

- Critically ill patients with acute kidney injury (AKI), especially those undergoing renal replacement therapy (RRT) in the intensive care unit (ICU), are at increased risk for new-onset atrial fibrillation (NOAF). Few data exist on the association between the timing of NOAF in relation to dialysis initiation and mortality in critically ill AKI patients undergoing RRT.
- We explored the incidence and associated outcomes of NOAF occurring before or after the initiation of RRT in critically ill AKI patients receiving continuous veno-venous haemodialysis or intermittent haemodialysis during ICU care.
- NOAF detected prior to RRT initiation in critically ill AKI patients during ICU stay was associated with increased mortality while NOAF occurring during RRT was not associated with mortality in this study.
- The timing of NOAF occurrence appears to have prognostic relevance in critically ill AKI patients receiving RRT irrespective of dialysis modality.

## Introduction

Atrial fibrillation (AF) is the most common arrhythmia in critically ill patients with an incidence of new-onset AF (NOAF) ranging from 4.5% in mixed medical and non-cardiac surgical patients, to as high as 29.5% after coronary artery bypass grafting.^1–3^ Furthermore, the risk for NOAF is higher in intensive care unit (ICU) patients undergoing renal replacement therapy (RRT) compared those with preserved kidney function.^4^ NOAF has been associated with increased mortality, morbidity, and hospital costs in both medical and surgical ICU patients.^5–7^ However, so far, only one study has addressed the incidence and outcomes of NOAF in critically ill ICU patients on RRT. Shawwa *et al.*^8^ explored the incidence of NOAF in critically ill ICU patients undergoing continuous renal replacement therapy (CRRT) and discovered a positive association between mortality and NOAF diagnosed after CRRT initiation, while patients observed with AF prior to CRRT initiation or undergoing intermittent haemodialysis (IHD) were not included in the study. Thus, there is lack of data on the incidence and subsequent outcomes of NOAF diagnosed in critically ill patients during ICU care before or after initiation of CRRT or IHD.

We sought to explore the incidence and determinants of NOAF in critically ill ICU patients undergoing continuous veno-venous haemodialysis (CVVHD) or IHD and to assess the effect of the timing of AF in relation to RRT initiation on 1-year all-cause mortality in a retrospective cohort study.

## Methods

**Figure 1:**
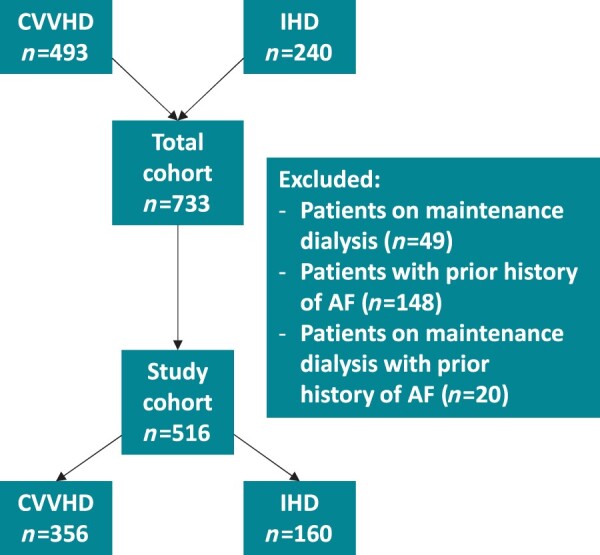
Flowchart of the study cohort.

This retrospective cohort study consecutively included all patients admitted to the ICU of the Turku University Hospital requiring CVVHD or IHD (733 participants) between 1 January 2010 and 31 December 2019. As the focus of this pre-specified report was the incidence and consequences of NOAF in critically ill acute kidney injury (AKI) patients requiring RRT, all patients on maintenance dialysis (49 patients) or with prior diagnosis of AF (148 patients) or both (20 patients) were excluded from the study. Prior diagnoses of AF were confirmed by ICD-10 code for AF (I48) and prior electronic ECG records. Thus, the final study cohort comprised 516 patients (*Figure 1*). No power calculations were performed due to the retrospective setting of the study.

All data on patient demographics, disease history, and medications at baseline were manually collected from the electronic patient records of the research hospital. Biochemical data at ICU admission and data on dialysis and ICU clinical parameters were extracted from the clinical information software of the research ICU. Incident NOAF episodes were collected from the pooled rhythm data of all the study patients recorded by the attending nurses and confirmed by the attending clinicians on site from continuous telemetry and/or ECG readings throughout ICU care. All patients were followed up from the electronic patient records to collect outcome data on 1-year mortality and cardiovascular embolic events within 3 years following ICU admission. Furthermore, data on mortality were collected from the national Digital and Population Data Services Agency directly linked to the patient archives to include mortalities outside the catchment area of the study hospital. Altogether 42 (8%) patients resided outside the healthcare district of the research hospital and hence were lacking data on cardiovascular embolic events. A cardiovascular embolic event was defined as an ischaemic stroke, a transient ischaemic attack (TIA), a limb embolus, or an intestinal embolus. The primary outcome measure of the study was all-cause mortality within 1-year follow-up.

### CVVHD, IHD, and sepsis

The choice of RRT modality between CVVHD and IHD was made according to clinical standards and based mainly on patient haemodynamics. CVVHD was primarily chosen for haemodynamically compromised patients.^9^

CVVHD for all patients receiving CRRT was performed according to a standard protocol employed in our centre using Fresenius Multifiltrate CRRT monitors and 1.80 m^2^ polysulfone haemofilter Ultraflux AV1000 or Ultraflux EMiC2 membranes with the CiCa^®^ dialysate K2 and 4% trisodium citrate to achieve regional citrate anticoagulation (Fresenius Medical Care, Bad Hamburg, Germany). Blood and dialysate flow rates were prescribed according to the weight of the patient and by the caring ICU physician to target a dialysis dose of 30 mL/kg/h.

IHD was performed using Fresenius Cordiax 5008 dialysis monitors with 2.5–5 h treatment duration, blood flow rates 170–300 mL/min, dialysate flow rate of 500 mL/min, and low-molecular-weight heparin anticoagulation dependent on the clinical condition of the patient and the running number of IHD treatment.

The presence of sepsis was defined as a suspected infection with a Sequential Organ Failure Assessment score ≥2.

### Ethics

The study protocol was approved by the Turku University Clinical Research Center scientific review board and the Hospital district of Southwest Finland (Reference number: T143/2019). The patient identity numbers were removed and the hospital software data combined before the statistical analyses. For this retrospective, register-based, non-interventional study the regulatory review board waived the need for informed consent in terms of data collection and analysis and publication of results.

#### Statistics

Results are reported as mean ± standard deviation (SD) for normally distributed covariates and as median (inter-quartile range) for skewed covariates unless stated otherwise. Categorical variables are presented with absolute and relative (percentage) frequencies. The Kolmogorov–Smirnov and Shapiro–Wilk tests were used to assess normality in continuous covariates. Comparisons for continuous normally distributed or skewed covariates were performed using Student’s *t*-test or Mann–Whitney *U* test as appropriate. Pearson *χ*^2^ or Fisher’s exact test was used to compare categorical covariates.

The univariate associations between the dependent variables and tested covariates were initially explored by entering the covariates separately into univariate logistic regression models. Due to the large number of tested variables, covariates included in the multivariable models were limited to those with univariate associations with the dependent variable at *P* ≤ 0.01 significance level.

The multivariable analyses were performed using logistic regression models to explore associations between tested covariates and incident NOAF during ICU care as well as all-cause mortality within the 1-year follow-up. As the relationship between incident NOAF and the time point of AF and all-cause mortality were the primary focus of the study, NOAF was entered as a three-level categorical variable (first level: no incident AF during ICU care, second level: incident NOAF observed after RRT initiation, and third level: incident NOAF observed prior to RRT initiation) in the multivariable logistic regression model. Dialysis modality, Acute Physiology And Chronic Health Evaluation II (APACHE II) score, prevalent mechanical ventilation and vasopressor support during ICU care were included as covariates in the multivariable model. Furthermore, the association between incident NOAF and all-cause mortality was explored in a similarly adjusted multivariable Cox proportional hazards model as well as in an unadjusted Kaplan–Meier time-to-event analysis with the use of a log-rank test. Potential existence of multicollinearity was assessed by examining variance inflation factors. Risk factors included in the APACHE II score such as age, haemodynamic parameters or chronic illnesses were not included separately in the multivariable model for 1-year mortality to avoid multicollinearity. Hosmer–Lemeshow goodness-of-fit test was performed for the multivariable models exploring the occurrence of NOAF and mortality with *P* > 0.60 for both models indicating good calibration.

Combined missingness in the multivariable models was <5%. Complete case approach was used. According to Little's test, the missingness in our current data was considered missing completely at random (*P* > 0.20) and thereby acceptable to employ a complete case analysis. The multivariable model for explanatory variables associated with incident AF included 493 patients (23 missing) and the multivariable model for 1-year mortality included 508 patients (8 missing).

All analyses were two-sided and *P* < 0.05 was considered statistically significant. IBM SPSS Statistics software version 26.0 was used to perform all analyses.

## Results

The median age of the study patients was 64 (52–71) years and 165 (32%) were female. The cause of ICU admission was medical in 347 (67%) and surgical in 169 (33%) patients and 56 (10.9%) patients underwent cardiac surgery. The index operation was elective in 30 (18%) of the 169 surgical patients. A total of 125 (24.2%) patients received treatment for sepsis during ICU care. The median duration of ICU stay was 5 (2–11) days and the median delay to RRT initiation was 0.6 (0.1–1.6) days from ICU admission.

Out of the 516 study patients, an incident NOAF was detected in 190 (37%) patients during ICU care and out of the 190 AF patients, 145 (28%), 38 (7%), and 7 (1%) patients were observed with one or more self-limited paroxysmal episodes of AF during ICU stay, an ongoing episode of AF at discharge from the ICU and persistent AF throughout ICU care, respectively. Moreover, 99 (19%) and 91 (18%) patients were observed with NOAF before and after RRT initiation, respectively. Patients diagnosed with NOAF before start of RRT were older and had higher APACHE II scores than those observed with NOAF during RRT (*Table 1*). Furthermore, 160 (45%) out of the 356 patients requiring CVVHD and 30 (19%) out of the 160 patients undergoing IHD were observed with NOAF. The baseline characteristics, medications, and blood biochemistry at ICU admission are summarized in *Table 2*. Older age, coronary artery disease, admission to the ICU due to cardiac surgery, higher SOFA score or Simplified Acute Physiology Score II or APACHE II score as well as need for mechanical ventilation or vasopressor support, CVVHD (vs. IHD), lower urine output, lower blood haemoglobin, lower blood thrombocyte count, lower plasma creatinine, or higher C-reactive protein were associated with the diagnosis of NOAF during ICU care in the univariate logistic regression analysis. In the multivariable logistic regression analysis, older age (OR 1.03 per year, 95% CI 1.02–1.05, *P* < 0.01), need for mechanical ventilation (OR 2.08, 95% CI 1.22–3.56, *P* < 0.01), CVVHD compared to IHD (OR 1.95, 95% CI 1.13–3.34, *P* = 0.01), and admission to the ICU due to cardiac surgery (OR 10.15, 95% CI 4.02–25.64, *P* < 0.01) were independently associated with NOAF during ICU care.

**Table 1:** Characteristics of patients observed with incident new-onset atrial fibrillation before and after renal replacement therapy initiation

Characteristics	Incident AF before RRT initiation (*N* = 99)	Incident AF after RRT initiation (*N* = 91)
Age mean (median), years	68 (71)	63 (64)
Female	33 (33)	25 (28)
Hypertension	65 (60)	48 (52)
Diabetes	33 (33)	24 (26)
Coronary artery disease	27 (27)	29 (32)
History of heart failure	14 (14)	15 (17)
Prior stroke	7 (7)	5 (6)
SOFA score	10 (7–12)	9 (7–13)
SAPS II score	56 (45–66)	55 (46–67)
APACHE II score	27 (22–32)	24 (20–29)
Mechanical ventilation	77 (78)	77 (85)
Vasopressor support	77 (78)	74 (81)
Dialysis—CVVHD (vs. IHD)	79 (80)	81 (89)
Urine output, mL/kg/h	0.4 (0.1–0.8)	0.1 (0.1–0.6)
Laboratory tests		
Haemoglobin, g/L	101 (90–113)	100 (88–115)
Thrombocytes, E9/L	135 (84–219)	114 (85–190)
C-reactive protein, mg/L	98 (29–243)	95 (44–228)
Sodium, mmol/L	137 (133–139)	137 (134–140)
Potassium, mmol/L	4.3 (3.9–4.9)	4.3 (3.9–5.0)
Creatinine, µmol/L	191 (116–299)	206 (106–329)
Ionized calcium, mmol/L	1.08 (1.01–1.14)	1.08 (0.98–1.13)
pH	7.29 (7.21–7.36)	7.30 (7.20–7.36)
Bicarbonate, mmol/L	17.8 (±5.3)	17.4 (±4.7)

Categorical values in parentheses are % unless stated otherwise. Continuous variables are expressed as mean (± SD) or median (IQR) for normally distributed and skewed covariates, respectively. The baseline laboratory values were measured at the time of ICU admission.

AF, atrial fibrillation; APACHE II score, Acute Physiology And Chronic Health Evaluation II score; CVVHD, continuous veno-venous haemodialysis; ICU, intensive care unit; IHD, intermittent haemodialysis; IQR, interquartile range; RRT, renal replacement therapy; SAPS II score, Simplified Acute Physiology II score; SD, standard deviation; SOFA score, Sequential Organ Failure Assessment score.

**Table 2:** Characteristics of patients with and without incident new-onset atrial fibrillation diagnosis during intensive care unit stay

Characteristics	Incident AF (*N* = 190)	No incident AF (*N* = 326)
Age mean (median), years	66 (67)	59 (62)
Female	58 (31)	107 (33)
Hypertension	113 (60)	168 (52)
Diabetes	57 (30)	97 (30)
Coronary artery disease	56 (30)	61 (19)
History of heart failure	29 (15)	43 (13)
Prior stroke	12 (6)	30 (9)
Treated for sepsis in the ICU	49 (25.8)	76 (23.4)
Admission to the ICU due to cardiac surgery	47 (24.7)	9 (2.8)
SOFA score	10 (7–12)	8 (5–11)
SAPS II score	57 (±17)	49 (±16)
APACHE II score	26 (21–31)	24 (18–29)
Mechanical ventilation	154 (81)	177 (54)
Vasopressor support	151 (80)	172 (53)
Dialysis—CVVHD (vs. IHD)	160 (84)	196 (60)
Urine output, mL/kg/h	0.3 (0.5–0.7)	0.3 (0.1–0.9)
Medications		
ACEi/ARB	73 (39)	119 (37)
Diuretic	56 (30)	76 (24)
Statin	62 (33)	92 (29)
Metformin	28 (15)	57 (18)
Insulin	27 (14)	39 (12)
NSAID	22 (12)	34 (11)
Laboratory tests		
Haemoglobin, g/L	101 (90–113)	109 (92–129)
Thrombocytes, E9/L	122 (85–204)	170 (108–248)
C-reactive protein, mg/L	98 (33–231)	59 (13–173)
Sodium, mmol/L	137 (134–140)	136 (132–140)
Potassium, mmol/L	4.3 (3.9–4.9)	4.4 (3.8–5.0)
Creatinine, µmol/L	202 (115–323)	242 (139–451)
Ionized calcium, mmol/L	1.08 (1.00–1.14)	1.06 (0.99–1.14)
pH	7.29 (7.20–7.36)	7.30 (7.19–7.37)
Bicarbonate, mmol/L	17.6 (±5.0)	17.0 (±5.5)

Categorical values in parentheses are % unless stated otherwise. Continuous variables are expressed as mean (± SD) or median (IQR) for normally distributed and skewed covariates, respectively. The baseline laboratory values were measured at the time of ICU admission.

ACEi, angiotensin-converting enzyme inhibitor; AF, atrial fibrillation; APACHE II score, Acute Physiology And Chronic Health Evaluation II score; ARB, angiotensin receptor blocker; CVVHD, continuous veno-venous haemodialysis; ICU, intensive care unit; IHD, intermittent haemodialysis; IQR, interquartile range; NSAID, non-steroidal anti-inflammatory drug; SAPS II score, Simplified Acute Physiology II score; SD, standard deviation; SOFA score, Sequential Organ Failure Assessment score.

**Figure 2:**
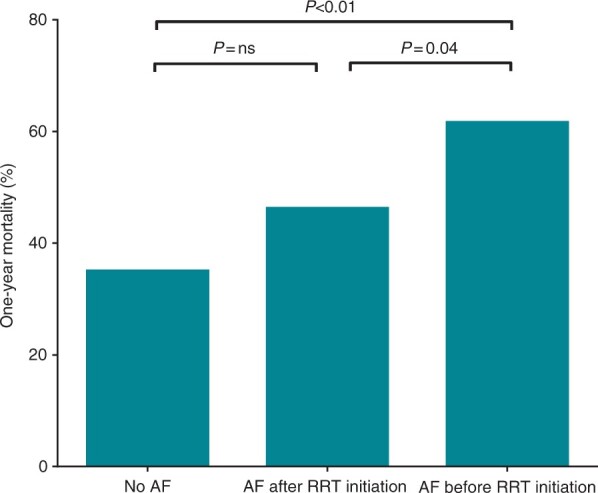
Comparison of all-cause mortality within 1-year follow-up between patients with no incident new-onset atrial fibrillation, incident new-onset atrial fibrillation after renal replacement therapy initiation, and incident new-onset atrial fibrillation before renal replacement therapy initiation during intensive care unit stay.

Altogether 217 (42%) patients died within the 1-year follow-up including 177 (50%) out of the 356 patients receiving CVVHD and 40 (25%) out of the 160 patients receiving IHD as the primary mode of RRT. The median time to death was 9 (2–36) days. A total of 127 (59%) patients perished during ICU care and 90-day mortality was 191 (88%) out of the 217 deceased patients. 72 (14%) patients died from cardiovascular causes. Incident NOAF was associated with all-cause 1-year mortality in univariate logistic regression analysis (OR 1.71, 95% CI 1.37–2.14, *P* < 0.01). The association between NOAF and mortality remained significant after adjusting for dialysis modality, need for mechanical ventilation, need for vasopressor support and APACHE II score in the multivariable logistic regression model (OR 1.41, 95% CI 1.10–1.80, *P* = 0.01). The multivariable findings were similar when a similarly adjusted Cox proportional hazards model was employed to study the association between NOAF and all-cause mortality (HR 1.21, 95% CI 1.03–1.43, *P* = 0.02). One-year mortality according to the timing of NOAF in relation to RRT initiation are depicted in *Figures 2 and* *3*. Mortality among patients observed with NOAF did not differ when comparing patients with one or more incident paroxysmal AF episodes, patients with ongoing AF at ICU discharge and patients with persistent AF throughout ICU care (data not shown). Furthermore, the association between NOAF and 1-year mortality remained significant (OR 1.51, 95% CI 1.16–1.95, *P* < 0.01) after the patients who died within 24 h after RRT initiation [48 (9%) patients] were excluded from the analysis.

**Figure 3:**
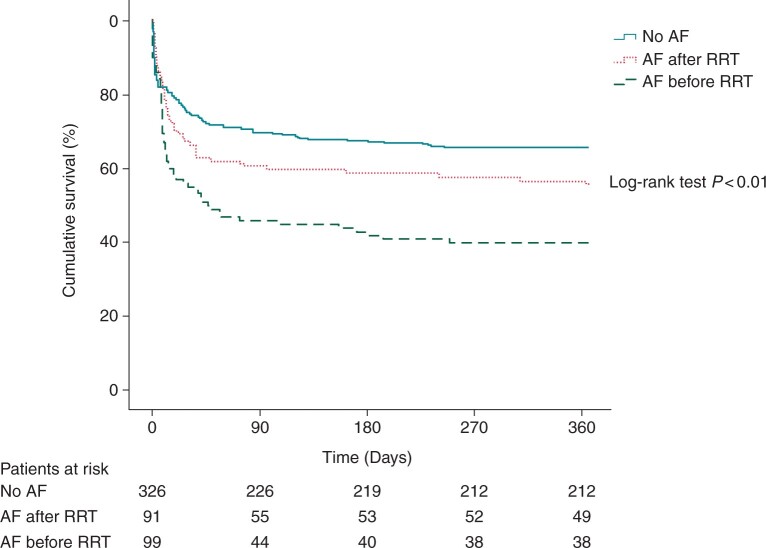
Kaplan–Meier survival curve for all-cause mortality within the 1-year follow-up according to the timing of incident new-onset atrial fibrillation during intensive care unit stay. AF, atrial fibrillation; CVVHD, continuous veno-venous haemodialysis; IHD, intermittent haemodialysis; RRT, renal replacement therapy.

Cardiovascular embolic events were observed within a 3-year follow-up in 18 (4%) patients. The embolic events included a stroke in 10 patients, a TIA in 3 patients, a lower limb embolic event in 2 patients, and an intestinal embolic event in 1 patient as well as 1 patient with a stroke and a TIA, separately, and 1 patient with a separate incident stroke and a lower limb embolic event. Out of the 18 patients observed with embolic events, 4 were receiving anticoagulation during the event. In univariate logistic regression analysis, female sex and need for mechanical ventilation were associated with an incident cardiovascular embolic event within the 3-year follow-up. In the multivariable logistic regression analysis, female sex (OR 4.23, 95% CI 1.54–11.56, *P* = 0.01) remained significantly associated with incident cardiovascular embolic event within follow-up. NOAF diagnosis regardless of timing in relation to RRT initiation during ICU care or rhythm status at discharge was not associated with cardiovascular embolic events.

## Discussion

Every third patient was observed with NOAF in this large retrospective cohort of mixed medical and surgical critically ill ICU patients undergoing RRT. NOAF was independently associated with all-cause mortality within 1-year follow-up after adjusting for disease severity in terms of APACHE II score, need for mechanical ventilation and vasopressor support and dialysis modality. Furthermore, the association remained significant when the patients who died within 24 h after RRT initiation were excluded from the analysis. However, NOAF emerging after RRT initiation was not associated with mortality (*Figure 2*). The incidence of cardiovascular embolic events was low despite high AF incidence in this study.

We demonstrate for the first time an association between NOAF observed prior to CVVHD or IHD initiation and 1-year all-cause mortality in critically ill patients requiring RRT. NOAF has been linked to increased mortality in critically ill patients in prior studies,^5–7^ while there are still very few data on the association between mortality and NOAF in those requiring RRT. Only a single previous study by Shawwa *et al*. recently explored the incidence and outcomes of NOAF after CRRT initiation in a cohort of 775 critically ill patients without prior AF history. They observed an NOAF incidence of 24.9% during CRRT and an increased risk for mortality in affected patients.^8^ Interestingly, and in contrast to their findings, we did not observe increased risk for 1-year mortality in patients with NOAF observed after RRT initiation regardless of dialysis modality. Notably, 1-year mortality was not reported in the reference study and NOAF was not associated with 180-day mortality in the whole cohort.^8^ Furthermore, in the study by Shawwa *et al*. NOAF data were compiled by collecting AF diagnoses recorded during ICU care in electronic patient records and confirmed with rhythm recordings if available, potentially leading to underdetection of NOAF. To avoid such bias, we screened all pooled rhythm recordings spanning the entire ICU stay of every patient for accurate detection of incident AF in the present study.

It is unclear why NOAF diagnosed before RRT initiation was associated with mortality whereas AF observed after start of RRT was not. The occurrence of NOAF in the critically ill has been associated with acute disease severity.^4^^,^^10^^,^^11^ Thus, NOAF prior to RRT initiation is probably an indication of higher morbidity and may carry more prognostic significance than AF onset after RRT initiation as RRT itself is a known trigger for AF.^10^^,^^12^ It is plausible that patients observed with NOAF before start of RRT were more susceptible to AF due to higher disease severity and more compromised haemodynamics and carried, thus, a poorer prognosis compared to patients observed with NOAF during RRT. Concordantly, the APACHE II scores were higher in patients observed with NOAF prior to RRT initiation compared with those with incident AF during RRT in the current study. However, the association between NOAF observed before RRT initiation and mortality remained significant after adjusting for dialysis modality, need for mechanical ventilation, vasopressor support, and APACHE II score. Therefore, it is possible that NOAF is not only a marker for disease severity in the critically ill but also a true risk factor for mortality in these patients. However, due to the retrospective study design causality cannot be determined from these data. The association between NOAF prior to RRT initiation (but not after RRT initiation) and 1-year mortality remained significant even after all the patients who perished within 24 h after RRT initiation were excluded from the analyses. This finding implies that NOAF may have long-term prognostic significance in patients with RRT-dependent AKI in addition to immediate adverse haemodynamic effects observed in prior studies on non-RRT ICU patients.^5^^,^^7^ In fact, it has been suggested that NOAF occurring during severe illness may predispose patients to further episodes and complications of AF and increased mortality in the following years.^13^ However, incident of NOAF after RRT initiation may be less significant in terms of mortality risk as it is often triggered by RRT and associated alterations in intravascular volume, acid–base balance, and potassium concentrations.^12^^,^^14^^,^^15^

The incidence of NOAF was high in the current study on mixed medical and surgical ICU patients compared to prior studies.^1–3^^,^^8^ Even though patients on maintenance dialysis were excluded, the high AF incidence is probably explained by the cohort selection as all patients required RRT for AKI—a risk factor for AF.^16^ In line with prior studies, older age and need for mechanical ventilation were independently associated with the occurrence of NOAF.^4^^,^^5^^,^^7^^,^^8^^,^^10^^,^^11^ Traditional risk factors for AF incidence such as hypertension and heart failure have performed poorly in critically ill patients in prior studies and our results did not differ from this pattern.^17^ Conversely, higher disease severity including need for mechanical ventilation and acute organ dysfunction have been associated with NOAF in critically ill patients.^10^^,^^18^ While RRT has been associated with an increased risk for NOAF during critical care,^4^ the risk for NOAF has not previously been compared in patients undergoing CRRT and IHD. In our study, the risk for developing NOAF was twice as high for patients receiving CVVHD as the primary RRT modality compared to those receiving IHD in the multivariable model. This is probably explained by patient selection as CRRT is considered the first-line choice for haemodynamically compromised patients.^19^

Cardiovascular embolic events were scarce in our study despite the extended 3-year follow-up and high comorbidity of the patients. The clinical practice for managing NOAF in critically ill patients especially in terms of anticoagulation is versatile due to the lack of data and international guidelines for critically ill AF patients.^20^^,^^21^ Prior data suggest that NOAF diagnosed during critical illness increases the risk for future stroke and further AF episodes.^13^ However, data on the benefit of oral anticoagulation for preventing later ischaemic strokes in patients observed with NOAF during critical illness remain inconclusive.^22^ Thus, further research is needed to determine the best course of action for reducing the risk of cardiovascular embolic events in patients with NOAF detected during critical illness.

Our study has all the limitations of an observational retrospective study. Due to the asymptomatic nature of AF, some short AF episodes may have been missed. However, we extensively screened all rhythm recordings collected by the attending ICU personnel for AF and observed a high incidence of AF suggesting good quality of AF data. As 42 (8%) patients had unavailable follow-up data on cardiovascular embolic events, we may have missed some embolic events. However, the majority (92%) of study patients resided in the catchment area of the study hospital and due to the overall low event rate, we believe the missing data to be negligible and not affect the primary conclusions. Despite these limitations, we believe that these data can be applied to clinical practice and guide future research.

## Conclusions

The incidence of NOAF was high in this large retrospective cohort study on critically ill ICU patients requiring RRT for AKI and a positive association between NOAF detected before RRT initiation and 1-year mortality was observed. However, NOAF observed after RRT initiation was not associated with mortality in this study.

**Conflict of interest:** none declared.

## Data availability

The datasets used and/or analysed during the current study are available from the corresponding author on reasonable request.
